# BMSeNet: Multiscale Context Pyramid Pooling and Spatial Detail Enhancement Network for Real-Time Semantic Segmentation

**DOI:** 10.3390/s24165145

**Published:** 2024-08-09

**Authors:** Shan Zhao, Xin Zhao, Zhanqiang Huo, Fukai Zhang

**Affiliations:** School of Software, Henan Polytechnic University, Jiaozuo 454000, China

**Keywords:** semantic segmentation, real-time, feature fusion, multiscale feature

## Abstract

Most real-time semantic segmentation networks use shallow architectures to achieve fast inference speeds. This approach, however, limits a network’s receptive field. Concurrently, feature information extraction is restricted to a single scale, which reduces the network’s ability to generalize and maintain robustness. Furthermore, loss of image spatial details negatively impacts segmentation accuracy. To address these limitations, this paper proposes a Multiscale Context Pyramid Pooling and Spatial Detail Enhancement Network (BMSeNet). First, to address the limitation of singular semantic feature scales, a Multiscale Context Pyramid Pooling Module (MSCPPM) is introduced. By leveraging various pooling operations, this module efficiently enlarges the receptive field and better aggregates multiscale contextual information. Moreover, a Spatial Detail Enhancement Module (SDEM) is designed, to effectively compensate for lost spatial detail information and significantly enhance the perception of spatial details. Finally, a Bilateral Attention Fusion Module (BAFM) is proposed. This module leverages pixel positional correlations to guide the network in assigning appropriate weights to the features extracted from the two branches, effectively merging the feature information of both branches. Extensive experiments were conducted on the Cityscapes and CamVid datasets. Experimental results show that the proposed BMSeNet achieves a good balance between inference speed and segmentation accuracy, outperforming some state-of-the-art real-time semantic segmentation methods.

## 1. Introduction

Semantic segmentation is a crucial computer vision task that attempts to obtain a comprehensive understanding of image content by accurately assigning each pixel in the image to specific semantic categories. This technology has broad application prospects in various fields, including human–computer interaction [1], autonomous driving [2,3], video surveillance [4], and medical image processing [5]. However, such application scenarios often require systems with fast inference speeds and high-precision semantic segmentation to realize real-time user interaction and response. Achieving this balance between fast inference speeds and high segmentation accuracy presents a significant challenge in real-time semantic segmentation. 

In recent years, significant advancements have been observed in semantic segmentation driven by fully convolutional network structure (FCN) [6]. Notably, FCN pioneered the use of a fully convolutional network structure to perform pixel-level classification of images. Following FCN, some exceptional semantic segmentation methods have been introduced. UNet [7] employs a U-shaped network structure and dense skip connections. This design allows UNet to retain intricate details during image processing and effectively capture global semantic information from images. SegNet [8] employs a symmetric encoder–decoder structure that recovers the encoder’s low-resolution feature maps while preserving maximal pool indexes and utilizing top–down lateral connectivity. This approach ensures the retention of complete information in feature maps. PSPNet [9] employs a pyramid pooling module that effectively extracts contextual information across various scales, which enhances the network’s ability to perceive global information. Chen et al. [10] utilized dilated convolutions to expand the network’s receptive field, allowing for extracting broader contextual information. In addition, Conditional Random Fields (CRF) were employed to improve the segmentation accuracy. DeepLabv3+ [11] is a design based on the pyramid structure dilated convolution, which utilizes dilated convolutions with different rates to expand the network’s receptive field while aggregating rich multiscale contextual information. HRNet [12] employs a multi-branch structure, with parallel branches introduced in the network to maintain high image resolution even as the network depth increases. Consequently, spatial detail and multiscale information are effectively extracted. DANet [13] leverages a spatial and channel attention module to capture global feature dependencies across both spatial and channel dimensions. CCNet [14] introduces a criss-cross attention module, which allows the extraction of cross feature information for each pixel across various directions and captures dense global contextual information. However, to enhance segmentation accuracy, high-precision semantic segmentation networks rely upon complex backbone networks such as ResNet [15] and VGG [16]. Although these complex backbone networks provide powerful feature representations, they also incur significant computational costs, which reduces inference speed. Furthermore, the primary focus of these high-precision networks is often to maximize segmentation accuracy, neglecting the equal importance of inference speed. As a result, they are unsuitable for applications that require real-time performance. 

To optimize inference speed, numerous real-time semantic segmentation networks prioritize lightweight networks as their backbone. This strategic choice aims to achieve a balance between efficient real-time execution and maintaining satisfactory segmentation accuracy without increasing computational costs. MobileNet [17], a lightweight classification network, has introduced a depth-wise separable convolutional structure. This ingenious design significantly reduces the network’s parameters and computational complexity, thereby facilitating an efficient inference speed while preserving satisfactory segmentation accuracy. ShuffleNet [18] is another notable lightweight classification network that employs shuffle operations to reduce the computational complexity, thereby enhancing the network’s computational efficiency. EfficientNet [19] employs a novel scaling method. This method employs a compound coefficient to strategically scale the network depth, width, and resolution in a balanced manner. This innovative approach optimizes the network performance while ensuring computational efficiency. However, lightweight classification networks often have limited feature extraction capabilities, potentially hindering the extraction of rich spatial and contextual information from the input image. Other authors [20,21], have explored reducing the size of the input images as a strategy to further improve inference speed. This is because adopting smaller input image resolutions significantly reduces computational complexity and inference time. However, lower resolutions can result in a significant loss of boundary information, which in turn reduces segmentation accuracy. The studies listed as references [8,22] increased the models’ inference speeds by decreasing channels in the networks because fewer channels translate to reduced computational costs, leading to faster inference speeds. However, reducing channels may weaken a model’s ability to represent spatial information.

In addition, various other notable network architectures have also emerged. ICNet [23] employs a three-level cascade architecture that effectively integrates detail information and semantic information. ESPNet [24] introduced an efficient spatial pyramid module that decomposes standard convolutions into a pointwise and spatial pyramid of dilated convolutions. This approach effectively reduces the model’s parameter count and computational complexity. CGNet [25] employs an effective context-guided block to efficiently model and extract global contextual information. Fast-SCNN [26] effectively extracts shallow features by employing two branches with shared learning for downsampling modules. BiSeNet [27] employs a dual-branch network architecture designed to preserve shallow spatial details while simultaneously extracting deep semantic information. The detail branch achieves this by extracting spatial detail information using shallow wide channels, and the semantic branch extracts semantic information via the design of deep narrow channels. BiSeNetV2 [28] also employs a dual-branch structure, where each path is dedicated to extracting detailed spatial information and categorical semantic information, respectively. STDC-Seg [29] builds upon BiSeNet [27] to achieve an improved balance between real-time inference speed and segmentation accuracy. The proposed network leverages a detail-guided approach to extract low-level detail information while minimizing computational costs. DDRNet [30] separates the backbone into two parallel depth branches. The first branch prioritizes the generation of high-resolution feature maps, and the second branch focuses on extracting semantic information through multiple downsampling layers. Effectively integrating information from both branches using multiple bilateral connections facilitates high performance in semantic segmentation tasks.

Image segmentation tasks are inherently complex because of the existence of multiple segmentation objects with different scales. This variability can result in different objects of the same category appearing in various sizes in the visual scene. During pixel-level classification in semantic segmentation, relying on a single scale makes it difficult for the model to accurately perceive targets of different sizes. This limitation reduces the network’s robustness and generalizability. Furthermore, the continuous downsampling of images during the segmentation process can lead to a significant loss of spatial detail information. To address the deficiencies in semantic feature diversity and spatial detail information loss in a real-time semantic segmentation network, this paper proposes a Multiscale Context Pyramid Pooling and Spatial Detail Enhancement Network (BMSeNet), which employs a dual-branch network structure. To address the issues of relatively singular semantic features, the proposed model incorporates a Multiscale Context Pyramid Pooling Module (MSCPPM). In addition, a Spatial Detail Enhancement Module (SDEM) was established to improve the extraction of spatial detail information. To seamlessly integrate spatial detail and semantic information, a Bilateral Attention Fusion Module (BAFM) was constructed. The primary contributions of this paper are as follows:

1. The design of the Multiscale Context Pyramid Pooling Module (MSCPPM) addresses the limited semantic feature diversity in segmentation tasks. The proposed MSCPPM realizes this by employing multiple pooling operations at different scales. This approach increases the network’s receptive field and facilitates the extraction of abundant local and global feature information from the input image. Consequently, it enables the capture of abundant multiscale contextual information, enhancing the model’s ability to perceive and process information at different scales.
2. In this paper, we introduce the Spatial Detail Enhancement Module (SDEM), which accurately acquires image position information through global average pooling operations in various directions. This module compensates for the loss of spatial detail information that occurs during the continuous downsampling process, thereby improving the model’s ability to effectively perceive spatial detail.
3. The Bilateral Attention Fusion Module (BAFM) effectively fuses spatial detail information and global contextual information. This is achieved by reasonably guiding the network to allocate weights based on the positional correlation between pixels in two branches, which improves the effectiveness of feature fusion.
4. To validate the efficacy of the proposed BMSeNet, comprehensive experiments were performed on two datasets. The results demonstrate that BMSeNet achieved 76.4% mIoU at 60.2 FPS on the Cityscapes test set, while on the CamVid test set, BMSeNet achieved 72.9% at 78 FPS.

## 2. Related Work

### 2.1. BiSeNet

**Figure 1:**
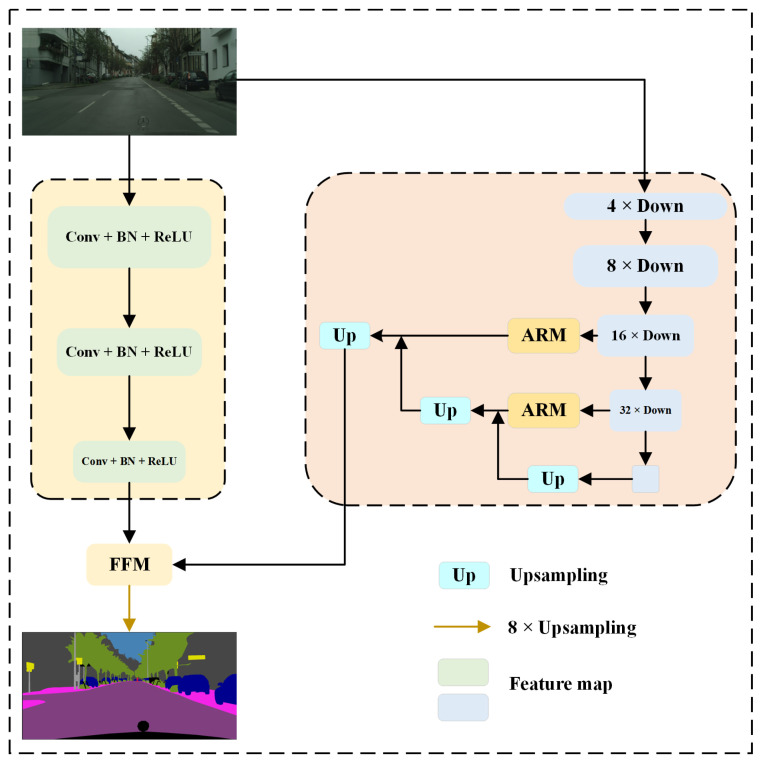
Overall architecture of BiSeNet.

The BiSeNet is a lightweight network model for real-time semantic segmentation. The overall structure is illustrated in Figure 1. In contrast to typical encoder–decoder architectures, BiSeNet employs a network structure with two branches. The dual-branch architecture in BiSeNet consists of two independent branches: the detail branch and the semantic branch, each responsible for different tasks. This design allows the network to retain shallow spatial details while simultaneously extracting deep global semantic information, resulting in a more comprehensive understanding of the image content. The detail branch concisely and efficiently extracts spatial details, generating high-resolution feature representations. Specifically, the detail branch comprises three Conv + BN + ReLU operations with a stride of 2, which generates feature map with rich spatial detail information at 1/8 the size of the original image. Rapid downsampling operations are employed by the semantic branch to acquire large receptive fields in the network, which facilitates the extraction of abundant contextual information. The semantic branch downsamples the input feature map by factors of 4, 8, 16, and 32, respectively, resulting in feature maps with sizes of 1/4, 1/8, 1/16, and 1/32 of the original feature map. The feature maps with significantly reduced sizes, 1/16 and 1/32 of the original size, are fed into the Attention Refinement Module (ARM) for further processing. Global average pooling is employed by the ARM to guide feature learning and refine feature representation, which enhances the understanding of semantic information in images. The feature map downsampled by a factor of 32 is fed into the global average pooling layer to increase the network’s receptive field. After refinement by the ARM, the feature maps undergo upsampling and addition operations to produce feature maps enriched with semantic information. The feature fusion module (FFM) acts as a bridge by merging the feature maps with detailed spatial detail information and those carrying rich semantic information from both branches. After merging, the fused features are upsampled by a factor of eight to return them to their original feature size. The FFM enhances feature fusion by applying weights across channels, which improves the integration of features from the two branches.

### 2.2. Attention Mechanism

The attention mechanism is extensively used in image segmentation. It learns the weight distribution across various image regions, allowing the model to selectively amplify or diminish the contribution of specific pixels. This enables the model to concentrate on critical information.

SENet [31] employs global average pooling and two fully connected layers to model the correlations between channels. By learning and adjusting the weight relationships between channels, it effectively weights the features of different channels. CBAM [32] focuses on both channel and spatial dimensions. The channel attention mechanism adaptively adjusts the weight of each channel, effectively capturing the correlation between channels. The spatial attention mechanism is employed to focus on significant regions of the image, thereby enhancing the perception of spatial structure. The attention mechanisms of these two dimensions are ultimately connected in series.

**Figure 2:**
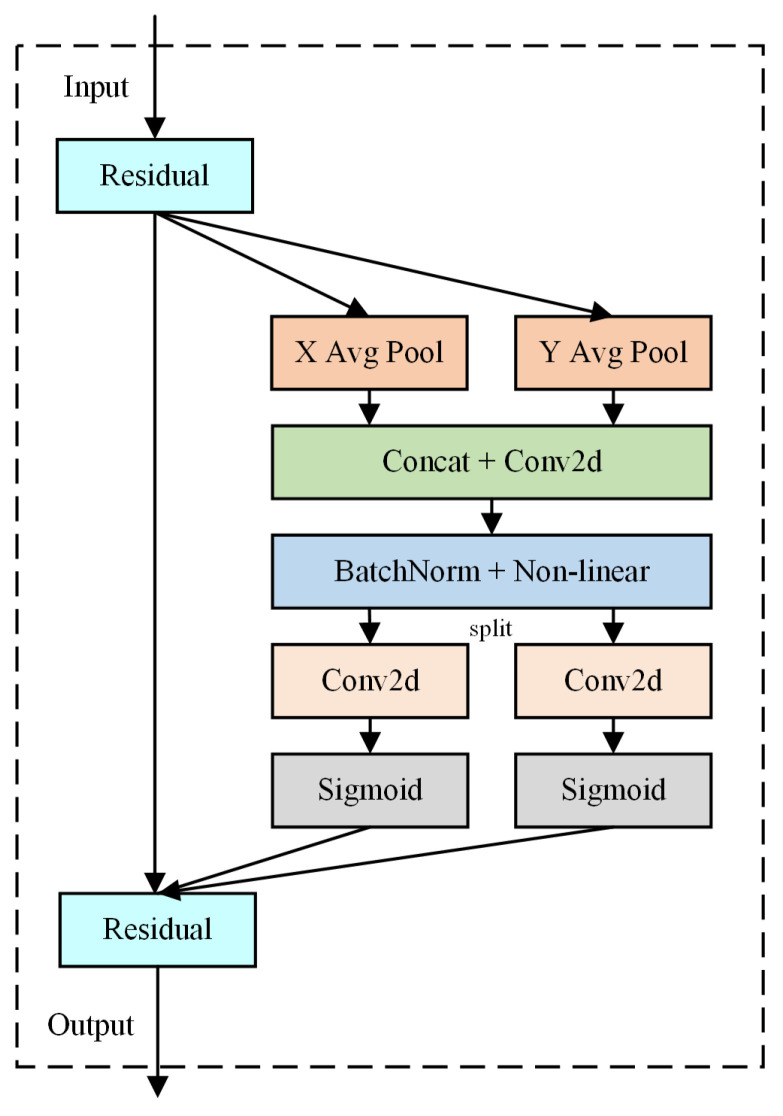
CA attention mechanism.

The Coordinate Attention (CA) [33] mechanism is a lightweight attention mechanism that combines channel information with positional details that are relevant to the direction. The proposed method focuses on channel-wise features while considering the position and orientation of these features in space. Figure 2 illustrates the CA structure. In CA, the initial step involves performing global average pooling operations in two distinct directions on the input feature map to derive separate feature maps for each direction. These feature maps are then concatenated to capture the positional information within the image. Next, the concatenated feature map undergoes shared 1 × 1 convolution, followed by batch normalization, and then it is split along the spatial dimension. Subsequently, the channel number is adjusted via 1 × 1 convolution, and horizontal and vertical attention weights are generated using the sigmoid function. Finally, the enhanced feature representation is obtained by multiplying the original feature map by the generated attention weights.

### 2.3. Feature Fusion

Image segmentation relies heavily on feature fusion, a technique that merges information from various levels or branches, thus improving overall segmentation performance. Recently, there has been a surge in innovative feature fusion modules designed to enhance the performance of semantic segmentation.

Attentional Feature Fusion (AFF) [34] utilizes attention mechanisms to assign weights to different features. This prioritizes informative parts of the image, ultimately capturing key details for segmentation tasks. PP-LiteSeg [35] introduced a Unified Attention Fusion Module (UAFM). This module utilizes channel or spatial attention mechanisms to improve the perception and expression of key features.

**Figure 3:**
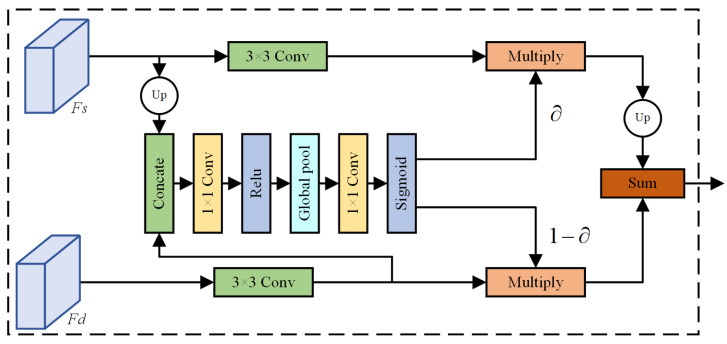
The architecture of AFM.

AttaNet [36] introduces an Attention Fusion Module (AFM) that significantly enhances overall segmentation performance. This is achieved by using a global attention mechanism to weight the importance of features at different hierarchical levels within the pixels, thereby obtaining multi-level feature representations. The AFM structure is illustrated in Figure 3. The process begins with two feature maps *Fs* and *Fd*. *Fs* is first upsampled to match the size of *Fd*. Simultaneously, *Fd* is processed through a 3 × 3 convolution for feature extraction. The extracted features are then concatenated with the upsampled features and input into a 1 × 1 convolution and ReLU activation function. Subsequently, relative attention weights are obtained through global average pooling, 1 × 1 convolution, and a sigmoid activation function. The calculated weights are subsequently applied to the features of each corresponding branch. The final step involves a pixel-by-pixel summation of the weights and features to obtain the final result.

## 3. Proposed Method

### 3.1. Overall Architecture

**Figure 4:**
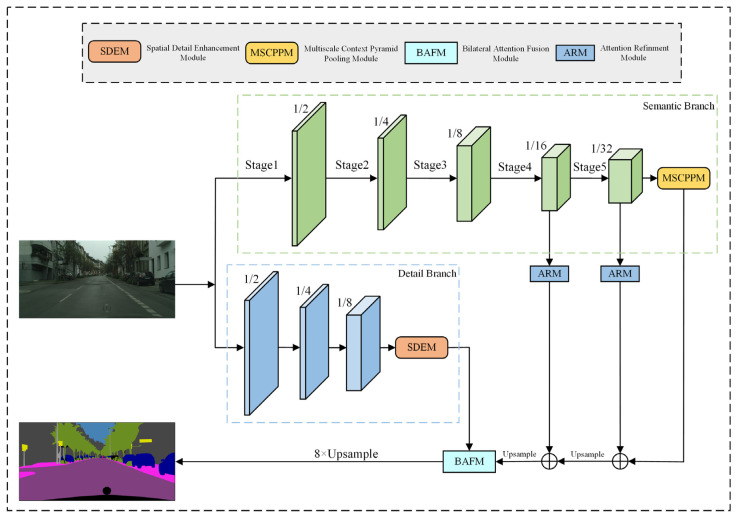
The architecture of our proposed BMSeNet.

Despite the impressive performance of BiSeNet-based segmentation networks, two pressing issues remain. First, relying on a single scale for semantic features reduces the network’s generalization and robustness. Second, there is a loss of spatial detail information, leading to a decrease in segmentation accuracy. To address these limitations, we propose a network called BMSeNet. The overall architecture of BMSeNet is depicted in Figure 4. As presented in Figure 4, BMSeNet follows a dual-branch network structure. The semantic branch comprising the MSCPPM and ResNet18 [15] backbone extracts rich semantic information. In contrast, the detail branch comprises the SDEM and spatial path from BiSeNet, which prioritizes extracting abundant spatial detail information. Beyond the input and prediction layers, the overall network architecture includes five stages, ranging from stage 1 to stage 5. Within each stage, BMSeNet employs a downsampling operation that reduces the input resolution by half using a stride of 2. A detailed explanation of the entire implementation process of BMSeNet is provided below.

BMSeNet begins by feeding the input image into its backbone network, which is designed to extract rich semantic information. After the image features pass through the backbone network, the MSCPPM is attached. The proposed method uses various pooling operations to enlarge the receptive field and efficiently gather diverse multiscale contextual information. In addition, the Attention Refinement Module (ARM) enhances the output features from the final two stages. After the three Conv + BN + ReLU operations, the Spatial Detail Enhancement Module is introduced to extract abundant spatial detail information, which enhances the model’s perception of spatial details. Then, the features extracted by both branches are combined through the BAFM. The BAFM seamlessly merges the informative details extracted by both branches, thereby improving the overall segmentation performance of the network. Following the BAFM, the resulting feature map is reduced to an eighth of its original size. Finally, the feature map undergoes an eightfold enlargement, bringing it back to its original size and enabling the final prediction.

Table 1 summarizes the detailed structure of BMSeNet. In this table, Dopr represents the convolution and DBEM operations in the detail branch, and Sopr encompasses the convolution, Max pooling, and MSCPPM operations in the semantic branch. Here, ‘C’ denotes the number of channels used at each stage, and ‘S’ represents the stride value employed during the downsampling process.

### 3.2. Multiscale Context Pyramid Pooling Module (MSCPPM)

In semantic segmentation, a critical component is the context aggregation module, which significantly improves segmentation performance. Moreover, by expanding the model’s receptive field, it becomes feasible to consider a broader range of contextual information and effectively grasp the long-range dependencies between pixels. This enhances the model’s global perceptual capability accordingly. At the same time, when dealing with images containing multiscale structures, the module can effectively perceive and capture the multiscale feature information present in the image. Processing images with closely related semantic associations enables accurate extraction of dense semantic relationships between different regions, which improves segmentation results.

**Figure 5:**
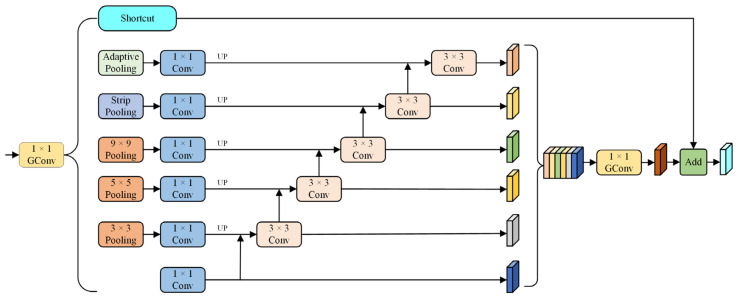
Architecture of the proposed MSCPPM.

MSCPPM is employed to enhance the accuracy of contextual information extraction from images. This allows for more effective extraction of rich multiscale contextual information, which improves the segmentation performance of the model. To ensure the inference speed of MSCPPM while enhancing accuracy, it is connected to the output feature map with a resolution of 1/32 of the original image of the backbone network. Since the input feature resolution of MSCPPM is only 1/32 of the original image resolution, the amount of computation is significantly reduced, resulting in a lower impact on inference speed. Additionally, 1 × 1 group convolution is used. By grouping the input feature map and performing independent convolution operations on each group, the number of parameters and computational complexity are reduced, thereby effectively improving the inference speed. The structure of MSCPMM is depicted in Figure 5. Within this module, the terms Pooling, Strip Pooling, UP, and GConv refer, respectively, to operations involving pooling, strip pooling, upsampling, and group convolution with a group size of 2. Initially, the input feature map undergoes a 1 × 1 group convolution to reduce dimensionality and improve computational efficiency. The resulting feature map undergoes a 1 × 1 convolution to generate a new feature map. Simultaneously, a set of pooling operations with kernel sizes {3 × 3, 5 × 5, 9 × 9} is applied. This is followed by 1 × 1 convolution and upsampling operations, which resize the feature maps to 1/32 of their original size from the input feature map. In addition, global contextual information is extracted by passing through a global average pooling layer in parallel, followed by 1 × 1 convolution and upsampling operations. At the same time, the model performs strip pooling operations in parallel, followed by 1 × 1 convolution and upsampling operations. This approach captures long-distance relationships within isolated regions while integrating both global and local contextual information. To maximize the utilization of feature information, the six aforementioned branches are connected in parallel. Each branch contributes sequentially to the next one, starting from the bottom. Feature information from various directions and scales seamlessly merges and interacts with the above operations. In addition, a 3 × 3 convolution is applied to fuse rich multiscale contextual information. Mathematically, if we represent the input features as $F_{x}$, the output features generated by each parallel branch of MSCPPM $Y_{i}$ can be expressed as follows:

(1) $$Y_{i}=\left\{\begin{array}{ll} C_{1\times 1}(F_{x}), & i=1;\\ C_{3\times 3}(Upsample(P_{pooling,j}(F_{x})))+Y_{i-1}), & 1<i<n-1,j=3,5,9;\\ C_{3\times 3}(Upsample(C_{1\times 1}(P_{\mathrm{global}}(F_{x})))+Y_{i-1}), & i=n-1;\\ C_{3\times 3}(Upsample(C_{1\times 1}(P_{\mathrm{strip}}(F_{x})))+Y_{i-1}), & i=n. \end{array}\right.$$

where $C_{1\times 1}$ represents the 1 × 1 convolution. $C_{3\times 3}$ represents the 3 × 3 convolution. Upsample indicates the upsampling operation. $P_{\mathrm{global}}$ expresses the global average pooling. $P_{\mathrm{strip}}$ signifies strip pooling. $P_{pooling,j}$ denotes a pooling operation with kernel size j.

Finally, the previously generated feature maps are combined via concatenation to form a unified feature map denoted as $F_{cat}$. After processing the concatenated feature map with a 1 × 1 GConv, it is connected residually to the feature map $F_{x}$, which proceeds into parallel branches. Then, the output feature $F_{out}$ of MSCPPM can be expressed as follows:

(2) $$F_{cat}=Concat(Y_{i})$$

(3) $$F_{out}=GConv(F_{cat})+F_{x}$$

where $F_{cat}$ represents the concatenated output features from the grounding operation of each branch $Y_{i}$, while GConv denotes group convolution.

### 3.3. Spatial Detail Enhancement Module (SDEM)

Spatial detail information is crucial in semantic segmentation and it encompasses specific local micro-features in the image, such as the position, size, and edges of objects. Simultaneously, preserving object boundaries and achieving accurate spatial positioning requires capturing essential spatial detail information. By accurately capturing spatial detail information, the model can effectively differentiate between various semantic categories and precisely delineate their boundaries, thereby enhancing its performance in complex scenes with numerous objects. Furthermore, the accurate acquisition of spatial details directly improves the overall segmentation performance, leading to higher-precision segmentation. 

**Figure 6:**
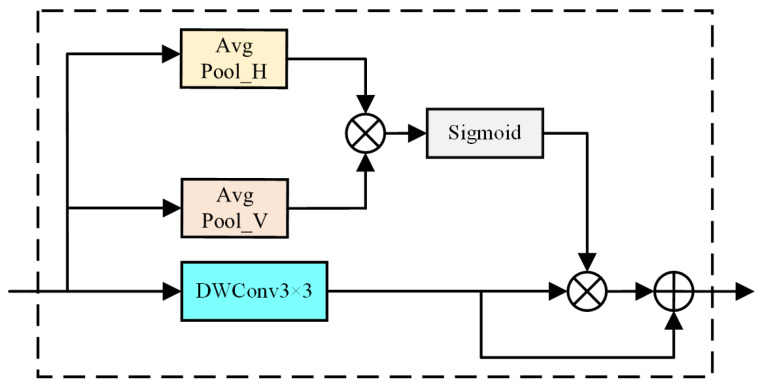
Architecture of the proposed SDEM.

To improve the accuracy of spatial detail information extracted from images, this study employs SDEM. We can extract spatial details from the image more effectively by accurately calculating the position of each object. This is achieved by multiplying the horizontal and vertical spatial features. In addition to improving accuracy, the inference speed of SDEM is considered. Specifically, 3 × 3 depth-wise separable convolution is employed. Depth-wise separable convolution reduces computational complexity and the number of parameters by decomposing the convolution into depth-wise and pointwise convolution. Compared to traditional convolution, depth-wise separable convolution provides higher computational efficiency, improving the model’s inference speed, Moreover, SDEM utilizes parallel computation by processing three distinct branches in parallel, further enhancing computational efficiency and accelerating inference. Figure 6 presents an overview of SDEM. This involves processing input features through three distinct branches: Depth-wise separable convolution enhances feature representation while reducing computational complexity and generates an enhanced feature map. Horizontal global pooling captures horizontal spatial features, and vertical global pooling captures vertical spatial features. In contrast to CA [33], which simply concatenates horizontal and vertical spatial features, SDEM leverages matrix multiplication between the horizontal and vertical spatial features, resulting in a feature map with positional information. The proposed approach enables better capture of pixel relationships across different locations, thereby enhancing its ability to effectively model spatial relationships. After capturing positional information within the feature map, a sigmoid function is applied to generate pixel-wise weights. These weights are then used to multiply the enhanced feature map, resulting in a feature map guided by these weights. Finally, the feature map guided by weights is added to the enhanced feature map element-wise to obtain the final output feature map of SDEM. This can be expressed mathematically as follows:

(4) ∂=Sigmoid[AvgPool_V(X)×AvgPool_H(X)]

(5) $$F_{out}=\partial \times DWC_{3\times 3}(X)+DWC_{3\times 3}(X)$$

where X represents the input to the SDEM, and the Sigmoid is denoted by the Sigmoid function operation. The output after processing through SDEM is indicated as $F_{out}$. The 3 × 3 depth-wise separable convolution operation is referred to as $DWC_{3\times 3}$. AvgPool_H signifies the global average pooling operation performed horizontally, whereas AvgPool_V denotes the global average pooling operation executed vertically.

### 3.4. Bilateral Attention Fusion Module (BAFM)

Feature fusion involves merging features from various levels or branches. The dual-branch network architecture leverages a feature fusion module to effectively integrate spatial detail information and semantic information. Spatial detail information and semantic information offer distinct types of insights. Local micro-features in the image, such as object position, shape, and edges, contribute to spatial detail information. High-level abstract information about the overall context of an image is included in the semantic information, which covers a global understanding of object categories, scene semantics, and the relationships between objects. Spatial detail and semantic information hold different levels of importance. Simply summing elements or concatenating channels is insufficient to effectively merge features from various levels. 

**Figure 7:**
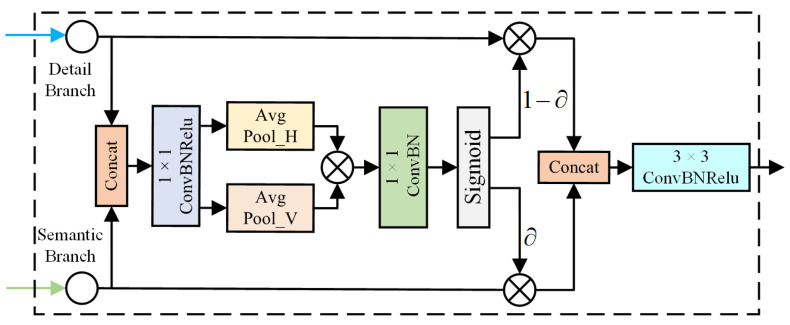
Architecture of proposed BAFM.

The BAFM is designed to facilitate a more effective integration of spatial feature information and semantic feature information extracted by the network’s dual branches. By considering the positional relationships between pixels, the model gains the ability to selectively focus on features from specific locations, thereby enhancing the recognition of critical areas and significantly enhancing the quality of feature fusion. BAFM also takes into account inference speed. It employs 1 × 1 convolution to reduce the number of channels in the fused feature map, thereby achieving dimensionality reduction. This effectively decreases the complexity of subsequent computations and enhances inference speed. Furthermore, BAFM synchronously processes feature information from both the semantic and detail branches. This enables the simultaneous utilization of features at different levels, reducing computational redundancy and further improving computational efficiency. As shown in Figure 7, the BAFM begins by concatenating the features $F_{s}$ and $F_{d}$ from the two branches. Next, to ensure a balance among features, BAFM applies a 1 × 1 convolution, batch normalization, and a ReLU activation function. The processed map is denoted as $F_{sd}$. Unlike AFM [36], which uses global average pooling, BAFM decomposes this step into horizontal and vertical global pooling. The proposed method allows the extraction of accurate spatial location information across extended spatial dependencies. The feature map $F_{sd}$ undergoes separate horizontal and vertical global pooling operations. This allows it to capture horizontal and vertical spatial features, respectively. The horizontal and vertical spatial features obtained are multiplied element-wise, producing a feature map with pixel-wise positional information. Subsequently, the feature map undergoes processing with a 1 × 1 convolution, batch normalization, and a sigmoid activation function to produce weights. Then, the weights are multiplied by each branch. Finally, the two generated features are concatenated. Further feature extraction is then conducted using a 3 × 3 convolution, batch normalization, and a ReLU activation function to derive the final output feature map $F_{out}$ of the BAFM. The detailed formulas for the entire process are as follows:

(6) $$F_{sd}=C_{1\times 1}(\mathrm{Concat}(F_{s},F_{d}))$$

(7) $$\partial =\mathrm{Sigmoid}\;[Conv((AvgPool\_V(F_{sd})\times AvgPool\_H(F_{sd})))]$$

(8) $$F_{out}=C_{3\times 3}(\mathrm{Concat}(F_{s}\times \partial ,F_{d}\times (1-\partial )))$$

where $C_{1\times 1}$ denotes the 1 × 1 convolution followed by batch normalization and the ReLU activation function. $C_{3\times 3}$ refers to the 3 × 3 convolution followed by batch normalization, and the ReLU activation function. Sigmoid indicates the use of the Sigmoid activation function. Conv refers to the 1 × 1 convolution followed by batch normalization. AvgPool_H denotes the global average pooling operation performed horizontally, while AvgPool_V denotes the global average pooling operation executed vertically.

## 4. Experiments 

This section begins with an introduction to the datasets used in our experiments, followed by a detailed explanation of the experimental settings. Subsequently, comprehensive ablation experiments are described to validate the efficacy of the designed modules in BMSeNet using the Cityscapes validation set. Lastly, our proposed method is benchmarked against other state-of-the-art approaches to validate its superiority.

### 4.1. Datasets

#### 4.1.1. Cityscapes 

Cityscapes [37] is an extensive dataset. It encompasses authentic urban street environments, showcasing a diverse array of city scenes populated with vehicles, pedestrians, buildings, and city streets. The dataset includes 5000 images with high-precision annotations, each featuring a resolution of 1024 × 2048. The data are partitioned into 2975 images for training, 500 images for validation, and 1525 images for testing. This dataset includes annotated images covering 30 distinct semantic categories, out of which 19 categories were specially employed for the semantic segmentation task.

#### 4.1.2. CamVid

CamVid [38] is a small-scale road scene segmentation dataset. Despite its limited size, this dataset remains one of the most commonly used datasets for real-time semantic segmentation applications. The dataset features 701 densely annotated frames. These frames, extracted from video sequences recorded on the streets of Cambridge, UK, showcase the complexity of road scenes. This dataset comprises 701 meticulously annotated images, each with a resolution of 720 × 960. These annotations categorize image elements into 32 distinct semantic classes, with 11 classes specifically used in the semantic segmentation task. The training set included 367 images, the test set included 101 images, and the validation set included 233 images.

### 4.2. Implementation Details and Evaluation

#### 4.2.1. Training Settings

Experiments were performed using PyTorch. During model training, the stochastic gradient descent (SGD) [39] algorithm was employed, with an initial momentum set to 0.9. In the experiment, we used the initial learning rate strategy where the initial rate was multiplied by ${\left(1-\frac{\mathrm{iter}}{\max\_i\mathrm{ter}}\right)}^{power}$, and the power was set to 0.9. For the first 1000 iterations, a linear warmup [40] was applied. For the Cityscapes dataset, we configured the batch size to 8, the initial learning rate to 0.01, applied weight decay to 0.0005, and trained the model for 80,000 iterations. We trained the model on the CamVid dataset using a batch size of 4, an initial learning rate of 0.01, a weight decay of 0.0002, and 20,000 iterations. The training process employs a single A100 GPU with CUDA 11.7, PyTorch 1.11, and cuDNN 8.0.

#### 4.2.2. Data Augmentation

This technique improves the model’s generalizability by augmenting data. We employed random cropping, scaling, and horizontal flipping for data augmentation during training. For the Cityscapes dataset, the cropping resolution was set to 1024 × 1024, while for the CamVid dataset, it was set to 720 × 960. The random scales comprised {0.5, 0.75, 1.0, 1.25, 1.5, 1.75}. The inference process involved image resizing to ensure compatibility with the model. Cityscapes images were resized to a resolution of 1024 × 1024, while CamVid images used 720 × 960. After making predictions, the results were adjusted to the original input size for the Cityscapes dataset.

#### 4.2.3. Evaluation

To assess the efficiency and performance of the proposed network, we employed three evaluation metrics: frames per second (FPS) to estimate computational complexity, mean intersection over union (mIoU) to quantify segmentation accuracy, and GFLOPs to estimate the computational complexity. Here is the formula used to calculate mIoU:

(9) $$mIoU=\frac{1}{k+1}\sum_{i=0}^{k}\frac{TP}{TP+FP+FN}$$

where TP stands for true positives, *FP* represents false positives, and FN denotes false negatives.

### 4.3. Ablation Study

In this section, ablation experiments were conducted to illustrate the efficacy of each component proposed in BMSeNet.

#### 4.3.1. Ablation and Comparison Analysis of MSCPPM

The effectiveness of the MSCPPM module in capturing multiscale contextual features was evaluated through ablation experiments in this section. To evaluate the effectiveness of MSCPPM against established methods, comparative experiments were conducted with several context aggregation methods. The results of the experiments are shown in Table 2.

Ablation Analysis: BiSeNet served as the baseline model. The global average pooling structure in the BiSeNet network was substituted with the MSCPPM design. The results presented in Table 2 demonstrated that integrating the proposed MSCPPM module increases the mIoU of the BiSeNet network from 74.40% to 75.82%, representing a 1.42% improvement.

Comparison Analysis: We explored how MSCPPM performs against other advanced context aggregation methods, such as DAPPM [30] and PAPPM [41]. Surpassing the BiSeNet network, the models demonstrated mIoU improvements of 1.42%, 0.80%, and 1.00%, respectively. Upon further analysis of the results from the ablation experiments, it becomes evident that the proposed MSCPPM design outperforms both DAPPM and PAPPM. The proposed MSCPPM achieves greater segmentation accuracy while introducing only minimal additional computational complexity.

**Figure 8:**
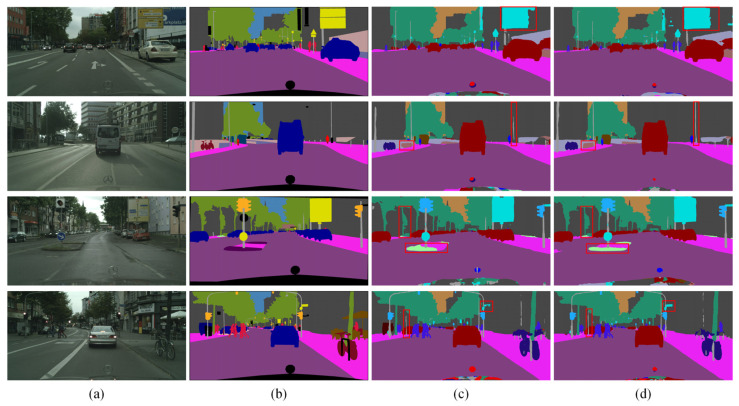
Ablation results for MSCPPM on the Cityscapes dataset: (**a**) original image, (**b**) mask image, (**c**) BiSeNet, and (**d**) BiSeNet + MSCPPM.

For a more lucid presentation of the ablation experiment outcomes, Figure 8 presents the visual results from four example images. Figure 8a presents the original image, while Figure 8b shows the mask image. Figure 8c,d demonstrate the segmentation predictions of BiSeNet and BiSeNet + MSCPPM, respectively. Notably, red boxes are used to emphasize the distinction between the two segmentation methods. The segmentation images generated by MSCPPM display enhanced completeness and refinement, i.e., they are characterized by sharper edges and richer details. This improvement stems from the MSCPPM’s ability to integrate diverse multiscale contextual information, enabling a more precise capture of semantic features within the images and thereby enhancing the overall segmentation quality. This underscores the effectiveness and superiority of MSCPPM.

#### 4.3.2. Ablation of SDEM

In this subsection, the significance of SDEM in extracting detailed spatial information is validated by conducting ablation experiments. Table 3 presents a comparison between the segmentation results of the baseline BiSeNet and the enhanced BiSeNet using SDEM.

As shown in Table 3, BiSeNet’s performance on the Cityscapes dataset achieved an mIoU of 74.40% with 55.46 GFLOPs. With the introduction of our designed SDEM, the mIoU improved to 75.34% with 55.76 GFLOPs. This represents a 0.94% increase in mIoU and a 0.30 GFLOPs increase in computational complexity. An analysis of the experimental results shows that incorporating SDEM significantly improves segmentation accuracy, while only marginally increasing the computational complexity. This suggests that SDEM is capable of effectively extracting abundant spatial detail information, leading to enhanced overall segmentation performance.

**Figure 9:**
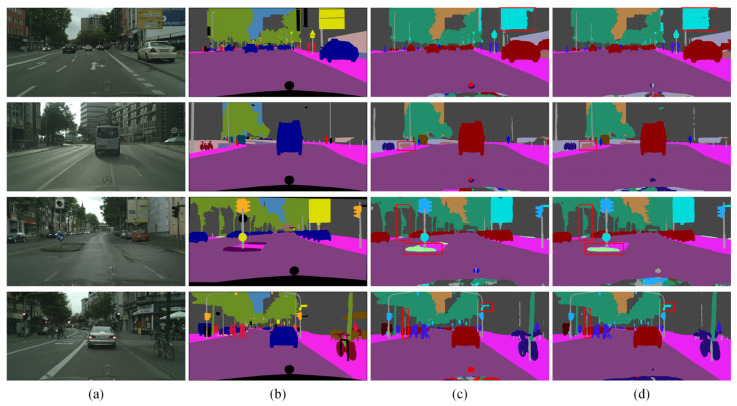
Ablation results for SDEM on the Cityscapes dataset: (**a**) original image, (**b**) mask image, (**c**) BiSeNet, and (**d**) BiSeNet + SDEM.

Figure 9 shows the visual results for four sample images from the Cityscapes dataset. In Figure 9a, the original images are displayed, while Figure 9b shows the corresponding mask images. Figure 9c,d present the prediction results, with Figure 9c showing the output from BiSeNet and Figure 9d showing the output from BiSeNet + SDEM. Figure 9 shows that edges segmented with SDEM display more refined features and effectively eliminate redundant edge information. This improvement stems from SDEM’s ability to capture positional information between pixels. It achieves this through horizontal and vertical pooling operations across the entire image, thereby allowing for a more precise understanding of edge location and shape. The visual results demonstrate the efficacy of SDEM.

#### 4.3.3. Ablation of BAFM

This section demonstrates the essential role of the BAFM in aggregating information from both branches, as confirmed by the ablation experiments. BiSeNet is the baseline model. We replaced its feature fusion module with our designed BAFM. To further validate the effectiveness of the BAFM, we compared both concatenation and addition operations. The experimental results are summarized in Table 4. In this table, FFM represents the feature fusion method used in the BiSeNet network, Concate denotes the concatenation operation, Add denotes the addition operation, and BAFM represents the proposed method.

Table 4 presents the segmentation accuracies, with mIoU scores of 74.40%, 74.03%, 74.05%, and 75.23% for the BiSeNet, Concate, Add, and BAFM methods, respectively. BAFM shows a 1.20% mIoU improvement over the Concate method, a 1.18% improvement over the Add method, and a 0.83% improvement over the FFM used in the BiSeNet network, with only a minor increase in computational complexity. These results indicate that BAFM effectively enhances the performance of dual-branch feature fusion.

**Figure 10:**
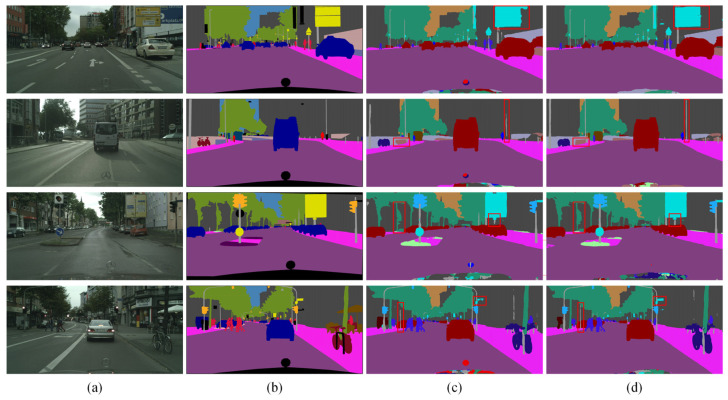
Ablation results for BAFM on the Cityscapes dataset: (**a**) original image, (**b**) mask image, (**c**) BiSeNet, and (**d**) BiSeNet + BAFM.

To further validate the experimental results, we provide visual examples of four images from the Cityscapes dataset, as illustrated in Figure 10. Figure 10a displays the original image, while Figure 10b shows the corresponding mask image. Figure 10c,d present the prediction results of BiSeNet and BiSeNet + BAFM, respectively. The red boxes highlight regions where the BAFM notably enhances segmentation accuracy. The results demonstrate that integrating the BAFM method leads to more precise segmentation results. In essence, the BAFM leverages positional correlations to guide the network in effectively allocating weights, which enhances the integration of feature information from both branches. These visual examples demonstrated how BAFM improves the aggregation of information from both branches.

#### 4.3.4. Ablation of Overall Architecture

To further validate the efficacy of the proposed module, the overall architecture of the network was evaluated on the Cityscapes dataset. The experimental findings are summarized in Table 5.

According to Table 5, the baseline network, BiSeNet, achieved a segmentation accuracy of 74.40% mIoU at 73.3 FPS. Introducing the MSCPPM module improved the segmentation accuracy to 75.82% mIoU, marking a 1.42% increase over the baseline network. This indicates that the proposed MSCPPM effectively aggregates rich multiscale contextual information, improving the segmentation performance of the model. Furthermore, with the addition of SDEM on this foundation, segmentation accuracy further improved to 76.27% mIoU, marking a 1.87% increase compared to the baseline network. This illustrates that SDEM effectively addresses the loss of spatial detail information, thereby enhancing the perception and integration of spatial detail. Finally, reintroducing BAFM as the feature fusion module slightly reduces the inference speed. However, the segmentation accuracy reached an mIoU of 76.90%, indicating a 2.50% increase compared to the baseline network. This suggests that BAFM effectively integrates shallow spatial and deep semantic information, thereby improving the model’s segmentation performance. The addition of the MSCPPM, SDEM, and BAFM modules significantly improves the model’s segmentation performance compared to the BiSeNet network. The experimental results confirm the effectiveness of the proposed modules, demonstrating substantial improvements in segmentation performance.

**Figure 11:**
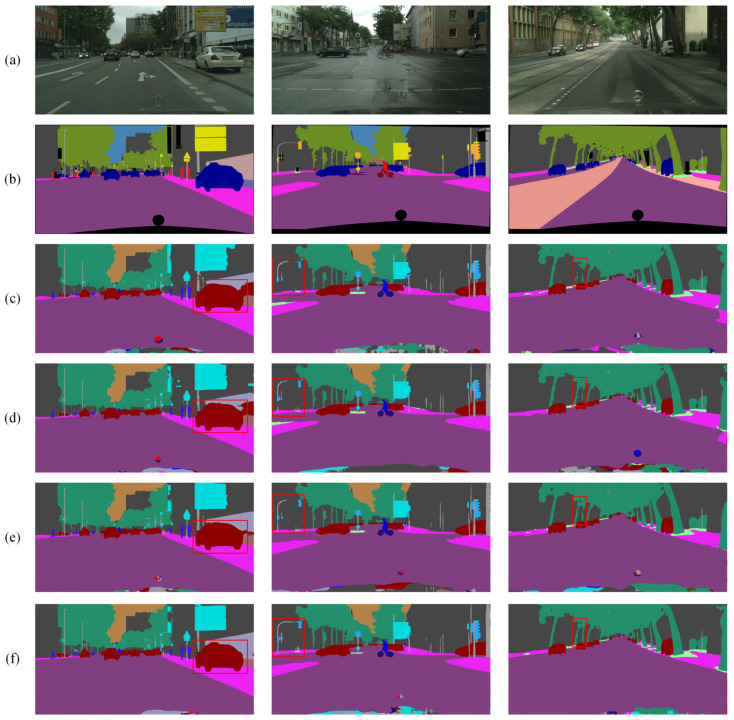
Ablation results for overall architecture on the Cityscapes dataset: (**a**) original image, (**b**) mask image, (**c**) BiSeNet, (**d**) BiSeNet + MSCPPM, (**e**) BiSeNet + MSCPMM + SDEM, and (**f**) BMSeNet.

To provide a clearer representation of the ablation study results, visual examples of three images are depicted in Figure 11.

### 4.4. Comparisons with State-of-the-Art Models

In this section, BMSeNet is assessed in comparison with state-of-the-art models on the Cityscapes and CamVid datasets.

#### 4.4.1. Comparison of Cityscapes Dataset

Table 6 presents a comparison of the results obtained by the proposed BMSeNet and other segmentation methods, including advanced real-time semantic segmentation algorithms and two common non-real-time semantic segmentation methods. Table 6 reveals that BMSeNet strikes a balance between inference speed and segmentation accuracy, achieving 76.9% mIoU and 76.4% mIoU at a speed of 60.2 FPS on the validation and test sets of Cityscapes, respectively. This method surpasses the previous DeepLab approach regarding inference speed and segmentation accuracy. On the test set, PSPNet achieved a segmentation accuracy of 81.2% mIoU; however, its low speed of 0.78 FPS renders it impractical for real-time applications. BMSeNet demonstrated superior segmentation accuracy compared to real-time methods. On the validation set, it surpassed STDC1-seg75 by an impressive 2.4% in mIoU, and on the test set, it outperformed HyperSeg-M by 0.6% in mIoU. Notably, BMSeNet achieved these improvements while running significantly faster, at a speed of 23.3 FPS. In comparison to BiSeNetV2, on the validation set, BMSeNet achieved a 3.5% higher mIoU. On the test set, BMSeNet achieved a 3.8% higher mIoU. Compared to SwiftNet, on the test set, BMSeNet achieved a 1.0% higher mIoU. In addition, BMSeNet outperformed CABiNet by 0.3% on the validation set. In summary, BMSeNet achieved superior accuracy compared to most real-time semantic segmentation algorithms, while maintaining a high inference speed.

**Figure 12:**
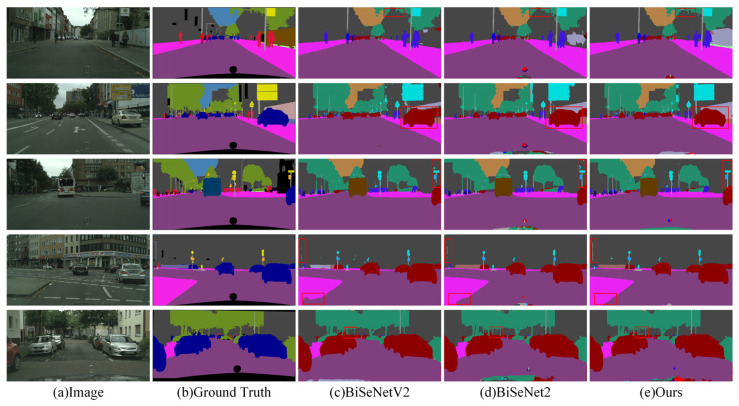
Visualization results obtained by different methods on the Cityscapes dataset.

The visual results obtained on the Cityscapes dataset are depicted in Figure 12, demonstrating three methods, including the model designed in this paper. Figure 12a represents the original image, Figure 12b displays the labels of the original image, and Figure 12c–e, respectively, show the segmentation results of BiSeNetV2, BiSeNet2, and the BMSeNet model designed in this paper. We emphasize the distinctions between various segmentation methods using red boxes. As shown in Figure 12, BMSeNet excelled in identifying categories such as vehicles, traffic signs, trees, and utility poles. Conversely, BiSeNetV2 and BiSeNet2 demonstrated relatively poorer performance in identifying these categories. This is primarily because MSCPPM effectively extracts rich multiscale contextual information, thereby performing exceptionally well in identifying less obvious categories. Furthermore, the BAFM efficiently merges spatial and semantic feature information, enhancing the model’s ability to perceive image details and thus enhancing the segmentation performance of the network.

#### 4.4.2. Comparison of CamVid Dataset

The proposed BMSeNet was compared with state-of-the-art methods on the CamVid dataset, further confirming its robustness and generalization. These methods encompass ENet [21], ICNet [23], LBN-AA [44], DFANet [43], BiSeNet1 [27], BiSeNet2 [27], S^2^-FPN18 [49], S^2^-FPN34 [49], BiSeNetV2 [28], TD^4^-Bise18 [45], and SwiftNet [46] as demonstrated in Table 7. According to Table 7, BMSeNet achieved 72.9% mIoU at 78 FPS on the CamVid test set. In comparison, BMSeNet surpassed BiSeNetV2, S^2^-FPN18, and ICNet by 0.5%, 3.4%, and 5.8% mIoU, respectively, in segmentation accuracy. In addition, BMSeNet achieved 1.3×, 2.8×, and 2.0× higher inference speeds than ENet, ICNet, and LBN-AA, respectively. These experimental results further substantiate the exceptional performance of BMSeNet using the CamVid dataset.

**Figure 13:**
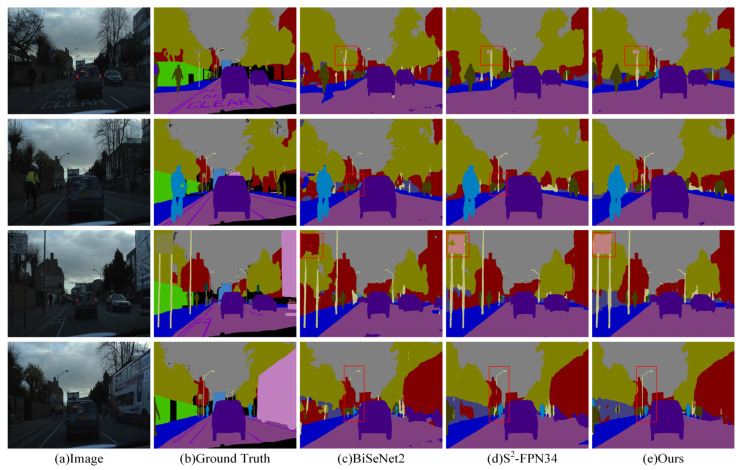
Visualization results obtained by different methods on the CamVid dataset.

The visual results of the three methods, including the model designed in this paper, on the CamVid dataset are depicted in Figure 13. Figure 13a shows the original image, while Figure 13b displays the corresponding labels. Figure 13c–e present the segmentation results obtained using three different models: BiSeNet2, S^2^-FPN34, and the proposed BMSeNet model. To better illustrate the differences between the segmentation methods, key differences were highlighted using red boxes. As shown in Figure 13, BMSeNet effectively identifies categories such as traffic signs and utility poles, while also demonstrating high object localization accuracy. The primary reason is that SDEM compensates for the loss of spatial detail information and enhances the model’s perception of spatial details, and thereby improves the overall segmentation performance. A review of the results of the experiments from the CamVid dataset shows that BMSeNet reaches a commendable balance between segmentation accuracy and inference speed.

## 5. Conclusions

This paper proposes BMSeNet, which is an improved semantic segmentation framework based on BiSeNet, for real-time semantic segmentation. BMSeNet incorporates a module called MSCPPM, which significantly improves the model’s ability to capture information at various scales. This module achieves this by expanding the receptive field of the network, which allows the network to effectively extract rich contextual details across different scales. Furthermore, SDEM was implemented to address the issue of spatial detail information loss. It not only compensates for this loss but also improves the perceptual sensitivity to spatial details. At the same time, the BAFM was incorporated. It was designed to efficiently merge information from both branches using positional correlations to guide the network in assigning appropriate weights to features, resulting in more accurate segmentation. The results of the experiments indicate that BMSeNet reaches a commendable balance between inference speed and accuracy. Future work will focus on optimizing BMSeNet components and exploring better information flow between branches.

## Figures and Tables

**Table 1:** Detailed structure of BMSeNet.

	Detail Branch			Semantic Branch			Output Size
	Dopr	C	S	Sopr	C	S	
Input							1024 × 1024
Stage1	Conv	64	2	Conv	64	2	512 × 512
Stage2	Conv	64	2	Maxpool	64	2	256 × 256
				Conv	64	1	
Stage3	Conv	64	2	Conv	128	2	128 × 128
	Conv	128	1	Conv	128	1	
	DBEM	128	-				
Stage4				Conv	256	2	64 × 64
				Conv	256	1	
Stage5				Conv	512	2	32 × 32
				Conv	512	1	
				MSCPPM	128	-	

**Table 2:** Ablation study of MSCPPM.

BiSeNet	DAPPM	PAPPM	MSCPPM	mIoU (%)	GFLOPs
✓				74.40	55.46
✓	✓			75.20	56.31
✓		✓		75.40	56.31
✓			✓	75.82	56.53

**Table 3:** Ablation study of SDEM.

Method	GFLOPs	mIoU (%)
BiSeNet	55.46	74.40
BiSeNet + SDEM	55.76	75.34

**Table 4:** Ablation study of BAFM.

Method	GFLOPs	mIoU (%)
BiSeNet	55.46	74.40
+Add	54.92	74.05
+Concate	54.37	74.03
+BAFM	56.07	75.23

**Table 5:** Ablation study of the overall architecture.

BiSeNet	MSCPPM	SDEM	BAFM	mIoU (%)	GFLOPs	FPS
✓				74.40	55.46	73.3
✓	✓			75.82	56.53	66.6
✓	✓	✓		76.27	56.83	62.2
✓	✓	✓	✓	76.90	57.44	60.2

**Table 6:** Comparison with state-of-the-art methods on the Cityscapes dataset.

Method	Backbone	GFLOPs	Val (%)	Test (%)	FPS
DeepLab [10]	VGG16	457.8	-	63.1	0.3
PSPNet [9]	ResNet101	412.2	-	81.2	0.78
ENet [21]	No	3.8	-	58.3	135.4
ICNet [23]	PSPNet50	28.3	-	69.5	30.3
ERFNet [42]	No	27.7	-	69.7	41.7
DFANet [43]	Xception A	3.4	-	71.3	100
LBN-AA [44]	MobileNetV2	49.5	-	73.6	51.0
BiSeNet1 [27]	Xception39	5.8	69.0	68.4	105.8
BiSeNet2 [27]	ResNet18	55.5	74.4	74.7	73.3
BiSeNetV2 [28]	No	21.1	73.4	72.6	156
TD^4^-Bise18 [45]	BiseNet18	-	75.0	74.9	47.6
SwiftNet [46]	ResNet18	104.0	75.5	75.4	39.9
STDC1-Seg75 [29]	STDC1	55.9	74.5	75.3	140.7
HyperSeg-M [47]	EfficientNet-B1	7.5	76.2	75.8	36.9
CABiNet [48]	MobileNetV3	12.0	76.6	75.9	76.5
BMSeNet	ResNet18	57.4	76.9	76.4	60.2

**Table 7:** Comparison with state-of-the-art methods on the CamVid dataset.

Method	Backbone	mIoU (%)	FPS
ENet [21]	No	51.3	61.2
ICNet [23]	PSPNet50	67.1	27.8
LBN-AA [44]	MobileNetV2	68.0	39.3
DFANet [43]	Xception A	64.7	120
BiSeNet1 [27]	Xception39	65.6	175
BiSeNet2 [27]	ResNet18	68.7	116.3
S^2^-FPN18 [49]	ResNet18	69.5	107
S^2^-FPN34 [49]	ResNet34	71.0	107.2
BiSeNetV2 [28]	No	72.4	124.5
TD^4^-Bise18 [45]	BiseNet18	72.6	25.0
SwiftNet [46]	ResNet18	72.6	-
BMSeNet	ResNet18	72.9	78

## Data Availability

The data presented in this study can be requested from the corresponding author, and these data are not currently available for public access.
